# The role of the radiologist in the evaluation of male infertility: recommendations of the European Society of Urogenital Radiology-Scrotal and Penile Imaging Working Group (ESUR-SPIWG) for scrotal imaging

**DOI:** 10.1007/s00330-024-10964-5

**Published:** 2024-07-31

**Authors:** Francesco Lotti, Michal Studniarek, Cristina Balasa, Jane Belfield, Pieter De Visschere, Simon Freeman, Oliwia Kozak, Karolina Markiet, Subramaniyan Ramanathan, Jonathan Richenberg, Mustafa Secil, Katarzyna Skrobisz, Athina C. Tsili, Michele Bertolotto, Laurence Rocher

**Affiliations:** 1 Department of Experimental and Clinical Biomedical Sciences “Mario Serio”, University of Florence, Florence, Italy; 2 Andrology, Female Endocrinology and Gender Incongruence Unit, University Hospital Careggi (AOUC), Florence, Italy; 3 Department of Radiology, Medical University of Gdańsk, Gdańsk, Poland; 4 Hôpitaux Paris Sud, Service de Radiologie Diagnostique et Interventionnelle, site Bicêtre, 94270 Le Kremlin Bicêtre, France; 5 Department of Radiology, Royal Liverpool University Hospital, Liverpool, UK; 6 Department of Radiology and Nuclear Medicine, Ghent University Hospital, Ghent, Belgium; 7 University Hospitals Plymouth NHS Trust, Derriford Hospital, Derriford Road, Crownhill, Plymouth, Devon PL6 8DH UK; 8 Department of Radiology, Al-Wakra Hospital, Hamad Medical Corporation, PO Box 82228 Doha, Qatar; 9 Department of Radiology, Weill Cornell Medical College, Doha, Qatar; 10 Department of Imaging, Brighton and Sussex University Hospitals NHS Trust and Brighton and Sussex Medical School, Brighton, UK; 11 Department of Radiology, Faculty of Medicine, Dokuz Eylul University, Izmir, Turkey; 12 Department of Clinical Radiology, School of Health Sciences, Faculty of Medicine, University of Ioannina, 45110 Ioannina, Greece; 13 Department of Radiology, University of Trieste, Ospedale di Cattinara, Trieste, Italy; 14 Hôpital Antoine Béclère, Service de Radiologie, APHP, 157 rue de la Porte de Trivaux, 92140 Clamart, France; 15 BIOMAPS. UMR1281. Université Paris Saclay, 63 Rue Gabriel Péri, 94270 Le Kremlin-Bicêtre, France

**Keywords:** Testicular ultrasonography, Epididymis and vas deferens ultrasonography, Varicocele, Male infertility, Testicular cancer

## Abstract

**Objectives:**

The Scrotal and Penile Imaging Working Group (SPIWG) of the European Society of Urogenital Radiology (ESUR) aimed to produce recommendations on the role of the radiologist in the evaluation of male infertility focused on scrotal imaging.

**Methods:**

The authors independently performed an extensive literature Medline search and a review of the clinical practice and consensus opinion of experts in the field.

**Results:**

Scrotal ultrasound (US) is useful in investigating male infertility. US abnormalities related to abnormal sperm parameters (sperm concentration, total count, motility, and morphology) are low testicular volume (TV), testicular inhomogeneity (TI), cryptorchidism, testicular microlithiasis (TML), high-grade varicocele, bilateral absence of vas deferens, bilateral dilation and echotexture abnormalities of the epididymis. The proposed ESUR-SPIWG recommendations for imaging in the evaluation of male infertility are therefore: to measure TV; investigate TI; perform annual (US) follow-ups up to age 55 in men with a history of cryptorchidism/orchidopexy and/or in men with TML plus “additional risk factors” or with “starry sky” TML; perform scrotal/inguinal US in men with nonpalpable testis; perform scrotal US in men with abnormal sperm parameters to investigate lesions suggestive of tumors; evaluate varicocele in a standardized way; evaluate the presence or absence of vas deferens; investigate the epididymis to detect indirect signs suggesting obstruction and/or inflammation.

**Conclusions:**

The ESUR-SPIWG recommends investigating infertile men with scrotal US focusing on TV, inhomogeneity, localization, varicocele, vas deferens, and epididymal abnormalities. Cryptorchidism, TML, and lesions should be detected in relation to the risk of testicular tumors.

**Clinical relevance statement:**

The ESUR-SPIWG recommendations on scrotal imaging in the assessment of male infertility are useful to standardize the US examination, focus on US abnormalities most associated with abnormal semen parameters in an evidence-based manner, and provide a standardized report to patients.

**Key Points:**

- *So far, ESUR-SPIWG recommendations on scrotal imaging in the assessment of male infertility were not available.*
- *The ESUR-SPIWG recommends investigating infertile men with scrotal US focusing on testicular volume, inhomogeneity, localization, varicocele, vas deferens and epididymal abnormalities, and assessing cryptorchidism, testicular microlithiasis and lesions in relation to the risk of testicular tumors.*
- *The ESUR-SPIWG recommendations on scrotal imaging in the assessment of male infertility are useful to standardize the US examination, focus on US abnormalities most associated with abnormal sperm parameters in an evidence-based manner, and provide a standardized report to patients.*

## Introduction

Male infertility affects up to 12% of men [1–3]. Despite technical advances, its etiology is still unknown in half of cases [1, 2]. The imaging of the male genital tract (MGT) has progressively expanded to improve diagnosis. Ultrasound (US) represents the gold-standard method for scrotal investigation [2, 4–7]. Scrotal US can assess features related to testicular damage, suggesting non-obstructive oligo-/azoo-spermia (NOA), or abnormalities at the epididymal and/or deferential level, suggesting obstructive oligo-/azoo-spermia (OA) [2, 4–7]. In addition, it can show features suggestive of testicular and epididymal inflammation and malignancy [2, 4–7]. The use of MGT imaging to investigate infertility is recommended by the European Academy of Andrology (EAA) [3–7], the European Association of Urology (EAU) [8], and the American Urological Association/American Society for Reproductive Medicine [9]. Based on a review of the literature and the practice of experts in the field, the aim of this study is to delineate the role of the radiologist in the evaluation of male infertility and establish the recommendations of the European Society of Urogenital Radiology-Scrotal and Penile Imaging Working Group (ESUR-SPIWG) for scrotal imaging.

## Methods

Guidelines were developed in accordance with the Appraisal of Guidelines for Research and Evaluation II document [10]. An extensive Medline search was performed by the ESUR-SPIWG members with no restrictions regarding the date of publication (i.e., from inception date until December 2023) including the following keywords: male infertility—scrotal ultrasound—testicular tumor—scrotal magnetic resonance imaging (MRI). Original and review articles as well as previous MGT imaging guidelines produced by international societies were considered, focusing on evidence-based studies. The identification of relevant studies in the English language was performed independently by all the authors. Consensus was obtained among the members of the ESUR-SPIWG. The quality of evidence was rated according to the Oxford Centre for Evidence-Based Medicine (OCEBM) 2011 levels of evidence (Supplementary Table 1) [11] and recommendations were graded using the Grading of Recommendations Assessment, Development, and Evaluation (GRADE) system (Supplementary Table 2) [12, 13]. The quality of evidence was classified into one of four levels: A: high quality; B: moderate quality; C: low quality; D: very low quality. The strength of the recommendations has been scored as “strong” or “weak”, depending on whether the quality of evidence in supporting it or not was graded A-B or C-D, respectively.

## Results

### Clinical investigation of male infertility

The investigation of male infertility includes personal and medical history, physical examination, semen analysis, hormonal parameters, and in specific cases, genetic investigation [3, 14, 15]. Table 1 shows the main aspects to evaluate and their relevance for male reproductive health [1–3, 14–18]. The radiologist should obtain infertility-related clinical data of the patient studied from the managing physician, and consider them to have an overall view of the case when performing the imaging investigation. The managing physician should get and deliver these data upon request.

**Table 1:** Clinical investigation of male infertility: what to assess and why?

What to investigate	Association with male infertility
Lifestyle	
Smoking habit	Negative effect on semen parameters [137], but no conclusions on male fertility reduction [138]
Alcohol consumption	Negative effect on semen volume [139, 140] and normal sperm morphology [140], but debated [141]
Cannabis consumption	Possible negative effect on male fertility [142, 143]
Physical activity	Recreational physical activity has a positive effect on sperm concentration and progressive motility [144]
Exposure to heat	Possible negative effect on male fertility [145]
Exposure to harmful substances/pollutants	Possible negative effect on male fertility [145]
Medical history	
Systemic diseases	Possible negative effect on male fertility [1]
History of cryptorchidism	Increased risk of infertility and testicular cancer [2, 7]
History of urogenital infections/inflammations	Debated effect on male fertility [2]
Past or current medications/therapies	Possible negative effect on male fertility [1]
History of testis trauma, torsion, tumor	Possible negative effect on male fertility [1]
History of surgery for inguinal hernia repair	Possible damage/obstruction of the vas deferens [2, 7]
Semen analysis	
Isolated sperm abnormalities	Suggest testicular dysfunction or bilateral epididymal (sub)obstruction [2]
Isolated low semen volume and pH	Suggest distal (sub)obstruction or seminal vesicles impairment/abnormalities/agenesis [2]
Sperm abnormalities and low semen volume and pH	Suggest distal (sub)obstruction [2]
Isolated azoospermia	Suggest testicular dysfunction including genetic abnormalities (karyotype or Y microdeletions) [2, 7] or bilateral epididymal/vas deferens obstruction [2, 7]
Azoospermia and low semen volume and pH	Suggest distal obstruction or bilateral vas deferens agenesis ± seminal vesicle/s agenesis/abnormalities (investigate *CFTR* mutations) [2, 7]
Unconventional semen parameters (e.g., sperm DNA fragmentation)	Possible negative effect on male fertility or increased risk of miscarriage [2]
Hormonal parameters	
FSH	High FSH levels ( > 8 U/L): tubular damage [3]
LH	High LH levels ( > 9.4 U/L): Leydig cells damage [3, 146]
Total testosterone (TT)	Low TT ( < 10.5 nmol/L): Leydig cells damage [3, 146]
SHBG	Evaluate SHBG when TT between 8–12 nmol/L, to calculate free testosterone (low when < 225 pM) [147]
Genetic tests	
Chromosomal abnormalities (karyotype)	Investigate when < 10 million spermatozoa/mL [148]
Y chromosome microdeletions	Investigate when < 5 million spermatozoa/mL [148]
* CFTR* gene mutations	Investigate when bilateral (or, rarely, unilateral) absence of vas deferens and/or seminal vesicles [148]

*FSH* follicle-stimulating hormone, *LH* luteinizing hormone, *SHBG* sex hormone binding globulin, *CFTR* cystic fibrosis transmembrane conductance regulator

*Recommendation 1: The radiologist should obtain infertility-related clinical data of the patient studied from the managing physician, who should get and deliver these data upon request*.

### What the radiologist should investigate and why?

The imaging of the scrotal region in investigating male infertility is mainly related to the assessment of (i) NOA, evaluating testicular abnormalities and varicocele, and (ii) OA, evaluating epididymal and vas deferens abnormalities [2, 4, 6, 7]. Table 2 summarizes what the radiologist should investigate and why. Table 3 summarizes the ESUR-SPIWG recommendations, reporting the level of evidence (LoE), grade (GoR), and strength of the recommendations. A standardized report is recommended (Table 4).

**Table 2:** What the radiologist should investigate and why

What to investigate?	Why?
Testis	
Volume	-Positive association with sperm parameters and testosterone, negative association with FSH and LH and unconventional sperm parameters (e.g., sperm DNA fragmentation) -Very small (and hard) bilateral testes (< 4 mL) (with high gonadotropins) suggestive of Klinefelter Syndrome -Small (and soft) testes (with low gonadotropins) suggestive of hypogonadotropic hypogonadism
Echotexture	-Testicular inhomogeneity associated with low sperm parameters and testosterone levels (non-obstructive infertility) -Rete testis dilation: suggestive of post-testicular obstruction -Multiple hypoechoic micronodules in Klinefelter Syndrome suggestive of Leydig cell hyperplasia
Masses/nodules	Vascularized solid or mixed nodules suggestive of tumors
Microlithiasis	-Likely association with infertility (debated) -Association with testicular tumor (especially in men with “additional risk factors”)
Localization	-Cryptorchidism or history of cryptorchidism/orchidopexy associated with low sperm parameters, testosterone levels, and risk of testicular tumor
Vascularization (low impact in the management of the infertile man)	-Absent: suggestive of testicular torsion (especially in men with pain) -Hypoechoic hypo-/a-vascular areas suggest previous testicular damage, with possible testicular impairment -Hyperemia: sign of current inflammation (orchitis), with a possible transient or permanent negative effect on sperm parameters
Stiffness (low impact in the management of the infertile man)	-Small and soft testes reflect parenchymal hypotrophy and impaired spermatogenesis. -Very small (< 4 mL) and hard symmetric testes suggest Klinefelter syndrome -Hard nodules suggest tumors
Varicocele	-Association with low sperm parameters (and testosterone levels), especially for high grades (IV–V) -Debated association with male infertility
Epididymis	
Dilation (and inhomogeneity)	-Suggestive of post-testicular (sub)obstruction (at epididymal, vas deferens (including CBAVD or CUAVD) or prostate level) with a possible negative effect on sperm parameters -Suggestive of past or current inflammation, with a possible negative effect on sperm parameters
Hyperemia	-Sign of current inflammation (epididymitis), with possible transient or permanent negative effect on sperm parameters
Absence	Associated with CBAVD with obstructive azoospermia, or CUAVD with normal or low sperm parameters
Vas deferens	
Dilation	-Suggestive of downstream (sub)obstruction (at vas deferens (e.g., retroperineal obstruction or vasectomy or surgical sequellae of hernia repair or absence of the distal part) or prostate level) with a possible negative effect on sperm parameters
Absence	Associated with CBAVD with obstructive azoospermia, or CUAVD with normal or low sperm parameters

For exhaustive details and references see the main text

**Table 3:** Summary of the ESUR-SPIWG recommendations on scrotal imaging in male infertility evaluation, with levels of evidence (LoE), grade (GoR), and strength of the recommendations

Recommendations		LoE	GoR	Strength
1	-The radiologist should obtain infertility-related clinical data of the patient studied from the managing physician, and consider them to have an overall view of the case when performing the imaging investigation.	LoE 5	GoR D	Weak
2	-Measure testicular volume (TV), since a low TV usually correlates with seminal and hormonal abnormalities, and report testicular diameters and mathematical formula used to calculate TV.	LoE 2	GoR A	Strong
	-The use of the ellipsoid formula (*V* = *L* × *W* × *H* × 0.52) is suggested.	LoE 5	GoR D	Weak
	-A right TV < 12 mL and/or a left TV < 11 mL indicate testicular hypotrophy.	LoE 2	GoR B	Strong
3	-Investigate testicular inhomogeneity, since it is usually associated with abnormal sperm parameters and low testosterone levels	LoE 2	GoR A	Strong
4	-Investigate TML for its likely association with infertility	LoE 3	GoR C	Weak
	-Investigate TML for its likely association with testicular cancer when “additional risk factors” are present or when a “starry sky” pattern is present	LoE 2	GoR A	Strong
	-Perform annual US follow-up up to age 55 in men with (i) TML and “additional risk factors” or (ii) “starry sky” TML.	LoE 3	GoR C	Weak
5	-Perform testicular US in men with a history of cryptorchidism due to the increased risk of infertility	LoE 2	GoR A	Strong
	-Perform testicular US in men with a history of cryptorchidism due to the increased risk of testicular tumor.	LoE 2	GoR A	Strong
	-US plays a key role in cancer detection or in the follow-up of the cryptorchid and contralateral testis.	LoE 2	GoR A	Strong
	-Perform annual US follow-up up to age 55.	LoE5	GoR D	Weak
6	-Perform scrotal/inguinal US in adult men with nonpalpable testis.	LoE 2	GoR A	Strong
	-If US is equivocal, inguinal/abdominal MRI or surgical exploration is advocated.	LoE 2	GoR A	Strong
7	-Perform testicular US in men with infertility to investigate testicular lesions suggestive of tumors, especially in men with oligo-/azoo-spermia or with risk factors for infertility and testicular tumor	LoE 2	GoR A	Strong
	-ESUR-SPIWG recommendations can be utilized to characterize nonpalpable lesions	LoE 4	GoR C	Weak
8	-The study of testis vascularization has no recognized impact on the clinical management of infertile men	LoE 2	GoR A	Strong
9	-The study of testicular stiffness with elastography has no recognized impact on the clinical management of infertile men	LoE 2	GoR A	Strong
10	-Varicocele evaluation is recommended in infertile men.	LoE 2	GoR B	Strong
	-Standardization of the US examination is essential.	LoR 1	GoR A	Strong
	-ESUR or EAA recommendations are suggested.	LoR 3	GoR C	Weak
11	-Testicular MRI is an emerging technique in male infertility evaluation, currently not recommended routinely.	LoE 4	GoR C	Weak
12	-Perform US evaluation for identification of CBAVD in men with OA.	LoE 2	GoR A	Strong
	-When CBAVD or CUAVD are detected, extend the US examination to the seminal vesicles and kidneys z(the latter especially for CUAVD).	LoE 2	GoR A	Strong
13	-Perform pelvic MRI when the US study of the vas deferens is doubtful/inconclusive or to evaluate the intra-abdominal course of the vas deferens,	LoE 2	GoR B	Strong
	-Perform pelvic MRI to investigate the prostate-vesicular region when suprapubic or transrectal US are doubtful/inconclusive assessing abnormalities related to suspected obstructive oligo-/azoo-spermia and/or low seminal volume and pH.	LoE 2	GoR B	Strong
14	-Perform US investigation of epididymis to detect indirect signs suggesting obstruction and/or inflammation, possibly exerting a negative impact on sperm parameters,	LoE 2	GoR A	Strong
	-Perform US investigation of epididymis to detect nodules suggesting tumors (usually benign).	LoE 5	GoR D	Weak
15	-In scrotal emergencies, the radiologist should evaluate the medical history and clinical signs and symptoms of the patient, and perform US to contribute to the diagnosis of testicular torsion, trauma, epididymo-orchitis or malignancy, which could exert a transient or long-lasting negative effect on sperm parameters and male fertility.	LoE 2	GoR B	Strong
	-In scrotal emergencies, scrotal MRI is rarely needed in cases of non-diagnostic US findings.	LoE 3	GoR C	Weak
16	-In infertile men, the radiologist should investigate the history of scrotal emergencies/acute scrotum to detect and/or understand related testicular US abnormalities.	LoE 5	GoR D	Weak

*LoE* levels of evidence, *GoR* grade of recommendation, *Strength* strength of the recommendation, *TV* testicular volume, *US* ultrasound, *TML* testicular microlithiasis, *OA* obstructive azoospermia, *CBAVD* congenital bilateral absence of the vas deferens, *CUAVD* congenital unilateral absence of the vas deferens. The quality of evidence was rated according to the Oxford Centre for Evidence-Based Medicine (OCEBM) 2011 levels of evidence (Supplementary Table 1) [11] and recommendations were graded using the Grading of Recommendations Assessment, Development and Evaluation (GRADE) system (Supplementary Table 2) [12, 13]. The quality of evidence was classified into one of four levels: A: high quality, B: moderate quality, C: low quality, and D: very low quality. The strength of the recommendations has been scored as “strong” or “weak”, depending on whether the quality of evidence in supporting it or not was graded A-B or C-D, respectively

**Table 4:** Example of a standardized US report for male infertility

Testis	R	L
Testicular localization (scrotal/high scrotal/inguinal/not found)		
Testicular diameters (L, W, H) in mm		
Testicular volume (report the mathematical formula used) in mL		
Testicular echotexture abnormality (Yes/No)		
Testicular homogeneity pattern (EAA classification (normal or mild/moderate/severe))		
Testicular echogenicity (mainly normoechoic, hypoechoic, hyperechoic)		
Testicular calcifications/microcalcifications/microlithiasis		
Testicular nodules/masses (number, size, vascularization, location)		
Testicular vascularization (present, diffusely or focally enhanced, or reduced/absent)		
Rete testis dilation (Yes/No)		
Hydrocele (Yes/No)		
Epididymis		
Presence/absence and measurement of head		
Presence/absence and measurement of body		
Presence/absence and measurement of tail		
Echotexture abnormalities (including tubular ectasia) (Yes/No)		
Vascularization (normal or enhanced)		
Cysts or solid nodules (Yes/No)		
Vas deferens		
Presence/absence^a^ and measurement when present		
Dilation/thickening		
Interruption/scar		
Varicocele		
Presence/absence		
Grading (ESUR [19, 20] or EAA [7] classification)		

^a^ If congenital bilateral absence of vas deferens (CBAVD) or congenital unilateral absence of vas deferens (CUAVD) are detected extend US examination to the seminal vesicles and kidneys^2^

#### Testis

The imaging of the testis should mainly focus on abnormalities of localization, volume, and echotexture, related to NOA, and findings increasing risk for malignancy [2, 4, 6, 7].

##### Testicular volume

Testicular volume (TV) should be measured as it usually correlates with testicular function [2, 4, 6, 7]. US-estimated TV is positively related to sperm parameters (sperm concentration, total count, motility, and normal morphology) and testosterone levels and negatively to FSH and LH levels and non-conventional sperm parameters (e.g. sperm DNA fragmentation) [2, 4, 6, 7]. TV reflects not only the seminal and hormonal status but also previous or current testicular or systemic disorders [2, 7]. Three different mathematical formulae can be used to calculate TV from US measurements of length (L), width (W), and height (H): ellipsoid’s, Lambert’s, and Hansen’s [2, 7]. The three diameters of the testis and the mathematical formula used to calculate TV should be reported. The ESUR-SPIWG guidelines on varicocele [19, 20] supported the use of Lambert’s formula (*V* = *L* × *W* × *H* × 0.71) according to some previous studies [21–23], however without “strong” consensus [19]. The EAA recently supported, in an evidence-based way, the ellipsoid formula (*V* = *L* × *W*  × *H* × 0.52) [4]. According to the EAA, the ellipsoid formula fits better with Prader orchidometer measurements and is easier to use in clinical practice since it is automatically calculated by most US devices [4, 6, 7]. The EAA US-TV lowest reference limit for right and left testis in healthy, fertile men, using the ellipsoid formula, is 12 and 11 mL, respectively, defining in an evidence-based manner the thresholds for “testicular hypotrophy” [4]. A normal TV does not exclude NOA, since patients with maturation arrest have often a normal TV [24]. Very small (< 4 mL) and hard symmetric testes associated with high gonadotropin levels suggest Klinefelter syndrome [2, 14, 25]. Small soft testes associated with low gonadotropin levels suggest hypogonadotropic hypogonadism [2, 14].

*Recommendation 2: Testicular volume (TV) should be assessed in men with infertility since a low TV usually correlates with seminal and hormonal abnormalities. The testicular diameters and mathematical formula used to calculate TV should be reported. The use of the ellipsoid formula is suggested. Right TV* < *12* *mL and left TV* < *11* *mL indicate testicular hypotrophy*.

##### Testicular echotexture

**Fig. 1:**
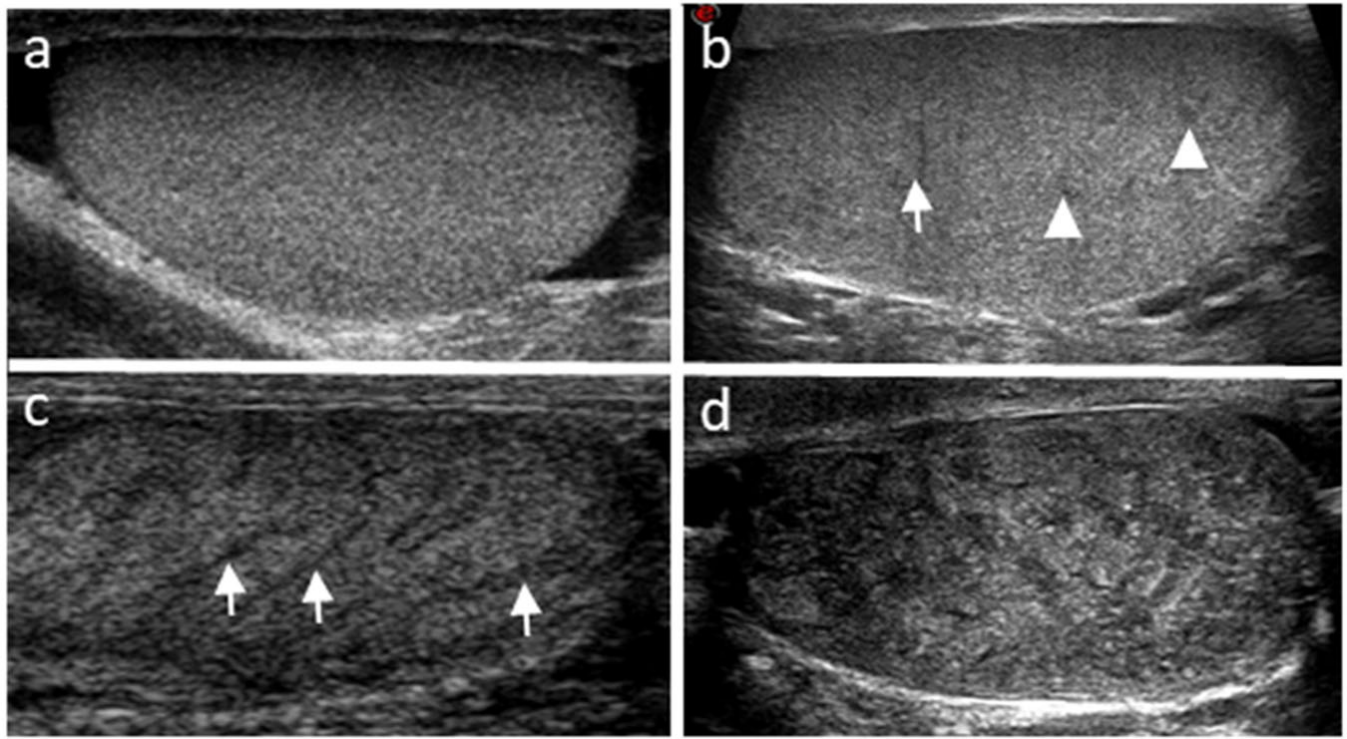
Testicular echotexture homogeneity classification of the EAA ultrasound consortium. **a** Homogeneity; **b** mild (grade 1) inhomogeneity: the presence of small hypoechoic foci (arrowheads)/thin hypoechoic striae (arrows); **c** moderate (grade 2) inhomogeneity: the presence of thick hypoechoic striae (arrows); **d** severe (grade 3) inhomogeneity: diffuse inhomogeneity with “netting”/“geographical map” appearance. Adapted from reference [4]

The normal adult testis is characterized by a homogeneous granular echotexture, made up of uniformly distributed medium-level echoes [2]. Echotexture alteration, especially testicular inhomogeneity (TI), frequently relates to testicular damage, abnormal sperm parameters, and low testosterone levels [2, 7]. At histology, TI reflects parenchymal atrophy and fibrosis [26]. TI has been detected in several conditions associated with male infertility, including cryptorchidism, affections leading to testicular damage, chemo- and radio-therapy [2, 26–29]. In addition, TI is frequent in Klinefelter syndrome, appearing as coarse or micronodular echotexture, with hypoechoic micronodules suggesting Leydig cell clusters/hyperplasia [30]. TI has been previously classified on a 5-point scale [31, 32] and, recently, on a 4-point scale by the EAA (Fig. 1), with higher scores suggesting more severe testicular damage [4]. On the other hand, rete testis dilation may suggest post-testicular obstruction [2].

*Recommendation 3: Testicular inhomogeneity (TI) should be investigated in men with infertility since it is usually associated with abnormal sperm parameters and low testosterone levels*.

##### Testicular microlithiasis (TML)

TML is an US diagnosis, commonly defined as the detection of ≥ 5 microcalcifications (bright echogenic non-shadowing foci < 3 mm) per field of view [2, 7, 33]. Its association with infertility and testicular cancer (TC) has been widely debated. Regarding infertility, several studies reported a higher TML prevalence in infertile than in fertile men [34, 35]. However, the association between TML and male infertility is still not universally recognized. Regarding TC, recent meta-analyses supported a significant association between TML and TC [36, 37]. However, recent reviews [34, 35] reported that TML is not an independent risk factor for TC, but is associated with TC when “additional risk factors” are present. According to the EAU guidelines [8, 38, 39], the presence of TML with “additional risk factors” (infertility, bilateral TML, atrophic testes, history of cryptorchidism, or TC) recommends scrotal US follow-up. The ESUR guidelines on TML imaging and follow-up [33] recommend annual US follow-up up to age 55 in patients with TML and “additional risk factors” (personal/family history of TC, maldescended testis, orchidopexy, testicular atrophy) and in men with diffuse (“starry sky”) TML.

*Recommendation 4: Testicular microlithiasis (TML) should be investigated in men with infertility. Its association with infertility is likely, but not universally recognized. TML is associated with TC, especially in men with “additional risk factors”. Annual US follow-up up to age 55 is advised in men with TML and “additional risk factors” or with “starry sky” TML*.

##### Cryptorchidism

Cryptorchidism is the absence of at least one testicle in the scrotum. It affects ~30% of premature infants, 3% of full-term babies, and ~1% of children in the third month of life [2, 40, 41]. However, its prevalence in men with severe infertility is almost 10% [42]. The undescended testis is commonly unilateral, being bilateral in 10% of cases [2, 40]. About 80% of undescended testes are located within the inguinal canal, 5–16% in the abdomen, while rarely the testis can be ectopic [2, 40, 41]. Cryptorchidism is associated with an increased risk of infertility [2, 40, 41, 43–46] and TC [2, 40, 41, 44, 47, 48]. TC commonly occurs in the undescended testis, however 20% of tumors occur in the contralateral descended testis [2, 40, 41]. A meta-analysis reported that the US does not reliably localize nonpalpable testes in pediatric patients [49]. Hence, all recent guidelines do not recommend the US in pediatric subjects [7]. However, guidelines on US in adult men with a history of cryptorchidism/orchidopexy are not available, despite the US playing a key role in cancer detection and follow-up of the cryptorchid and contralateral testis [2, 7]. Since cryptorchidism is a greater risk factor for TC than TML, recommendations given by the ESUR for TML [33] could be extended to men with a history of cryptorchidism/orchidopexy, i.e. annual follow-up up to age 55.

In addition, some men may present with a nonpalpable testis. Since US can reliably identify a cryptorchid testis lying below the internal inguinal ring [50], US may be suggested to identify the undescended testis in the inguinal canal or in the upper scrotum. If US is inconclusive, inguinal/abdominal MRI or surgical exploration should be suggested [7, 51]. At US, the cryptorchid testis is often hypotrophic, inhomogeneous, and hypoechoic, with calcifications [7, 51, 52]. Nodular lesions may be present [7, 51, 52] and should be managed according to available guidelines [38, 53].

*Recommendation 5: Testicular US is recommended in men with cryptorchidism or a history of cryptorchidism/orchidopexy. Cryptorchidism is associated with an increased risk of infertility and TC. US plays a key role in cancer detection or follow-up of the cryptorchid and contralateral testis. The ESUR-SPIWG suggests annual US follow-up up to age 55*.

*Recommendation 6: In adult men with nonpalpable testis, US is suggested to identify the undescended testis in the inguinal canal or upper scrotum. If US is inconclusive, inguinal/abdominal MRI or surgical exploration is advocated*.

##### Cancer risk

Male infertility is associated with an increased risk of TC. Men with abnormal sperm parameters are at an increased risk of TC, the worse the parameters the greater the risk [54, 55]. In infertile men, a history of cryptorchidism [2, 40, 41, 44, 47, 48] or the presence of TML [36, 37] is associated with an increased risk of TC. Infertile men have a higher prevalence than fertile men of cryptorchidism [42] and TML [34, 35], associated with an increased risk of TC. Men with TC show lower semen quality compared to men without TC [56]. Men with TC are frequently azoo-/oligo-spermic [57–59].

The scrotal US in male infertility screening reveals testicular lesions in 2–4% of cases [60]. Most of these lesions are nonpalpable and represent a clinical challenge [61–64]. However, the majority of nonpalpable lesions are non-malignant [65]. ESUR-SPIWG recommendations regarding incidentally detected nonpalpable testicular tumors in adults are available [53]. Multiparametric US can help in the characterization of doubtful lesions [66, 67].

*Recommendation 7: Testicular US is recommended in men with infertility to investigate testicular lesions suggestive of TC, especially in subjects with oligo-/azoo-spermia or with risk factors for infertility and TC. ESUR-SPIWG recommendations can be utilized to characterize nonpalpable lesions*.

##### Testicular vascularization

Studies focused on testicular vascularization and male infertility are scanty [68–72]. Some vascular parameters have been associated with sperm quality [73, 74] or discrimination of OA and NOA [68, 72, 75, 76]. However, at present, testicular vascularization has no impact on the clinical management of infertile men. Of note, recently the EAA reported a standardization of the measurement of testis vascular parameters and their reference ranges in healthy, fertile men [4].

Diffuse or focal hypoechoic hypo-/a-vascular testicular areas can suggest previous testicular damage, as previous testicular torsion, trauma, inflammation, lobular ischemia, or testicular sperm extraction [2, 7], which can be associated with impaired testicular function.

*Recommendation 8: The study of testis vascularization has no recognized impact on the clinical management of infertile men*.

##### Testicular stiffness

Evaluation of testicular stiffness by digital palpation is a clinical sign usually checked in infertile men. Small and soft testes suggest parenchymal hypotrophy and impaired spermatogenesis [2, 10]. Very small (< 4 mL) and hard symmetric testes suggest Klinefelter syndrome [2, 10, 25]. Two US approaches are available to evaluate testicular stiffness: Strain and Shear-Wave Elastography. A few studies focused on elastography and infertility, to distinguish obstructive and non-obstructive patterns, with disappointing results [77, 78].

*Recommendation 9: The study of testicular stiffness with elastography has no recognized impact on the clinical management of infertile men*.

##### Varicocele

Varicocele represents a common co-factor of male infertility [79]. It is more prevalent in infertile than fertile men [80] and has been associated with testicular damage and impaired spermatogenesis [81–83]. However, many men with varicocele have normal sperm parameters and are fertile [4, 7, 83]. Hence, the effect of varicocele on male fertility is debated and, so far, international societies support surgical correction only in highly selected cases [8, 9]. Physical examination has low accuracy for detecting varicocele in comparison with US [84], which is the imaging modality of choice. US is useful to assess varicocele when the clinical examination is unreliable, to grade varicocele, and to detect “false” clinical varicocele and post-operative recurrence/persistence [2, 85].

Evidence-based recommendations for standardization of the US examination have been published by the ESUR-SPIWG [19, 20] and the EAA [4, 7], and are very similar. These recommendations emphasize the importance of a standardized examination technique and provide diagnostic criteria [86–89].

*Recommendation 10: Varicocele evaluation is recommended in infertile men. Standardization of the US examination is essential. ESUR or EAA recommendations are suggested*.

##### Testicular MRI

At present, testicular MRI has no established role in the routine work-up of male infertility. However, advancements in functional MRI techniques [90–108], including diffusion-weighted imaging [90–98], volumetric apparent diffusion coefficient histogram analysis [99], diffusion tensor imaging [100–102], magnetization transfer imaging [94, 96] and proton MR spectroscopy [97, 103, 107] might provide novel insights in the future. Recent studies reported the ability of these techniques to distinguish OA and NOA [95, 97, 98], identify NOA etiology [108], assess early indicators of impaired spermatogenesis [90–94, 97, 105], and predict the surgical recovery of spermatozoa in NOA [95–99, 101–104, 106, 107]. However, due to the need for more, strong, evidence, and the high cost of the exam, currently, testicular MRI cannot be recommended routinely. Of note, MRI is useful in the characterization of testicular lesions doubtful in US [109].

*Recommendation 11: Testicular MRI is an emerging technique in male infertility evaluation, currently not recommended routinely*.

#### Epididymis and vas deferens

Evaluation of epididymis and vas deferens is useful in distinguishing OA and NOA [2, 7]. In particular, congenital bilateral absence of vas deferens (CBAVD) or bilateral epididymal obstruction are associated with OA [2, 7]. Scrotal US is the gold standard for evaluating the epididymis and vas deferens [2, 7]. Recently, the EAA reported a standardization of the measurements, and identified reference ranges and normative thresholds, for epididymal segments and vas deferens size and vascular parameters [4]. Normal epididymal head, body, tail, and vas deferens have been defined in an evidence-based way as < 11.5, 5, 6, and 4.5 mm, respectively [4, 7].

##### Vas deferens

The detection of CBAVD leads to a proven diagnosis of OA. CBAVD is present in 1–2% of infertile men and 4–17% of azoospermic men [106]. Since CBAVD is frequently associated with seminal vesicle (SV) agenesis [110, 111], azoospermia is often associated with low seminal volume and pH, and the US examination should be extended to the prostate-vesicular region [2]. Since CBAVD is usually associated with the *CFTR* (Cystic Fibrosis Transmembrane Conductance Regulator) gene mutation [112], genetic counseling should be recommended. CBAVD men usually show normal TV and testicular function, hence after CBAVD detection testicular sperm extraction can be indicated [2].

Scrotal US can also detect congenital unilateral absence of the vas deferens (CUAVD). This condition is present in 1% of infertile men [106], although men with CUAVD can show normal sperm parameters and be fertile [2]. Since CUAVD is frequently associated with ipsilateral SV agenesis [106], men may present with low seminal volume and pH, and the US examination should be extended to the prostate-vesicular region [2]. Since CUAVD is frequently associated with ipsilateral kidney agenesis (rare in CBAVD patients) [110, 111], the US examination should be extended to the abdominal region. Finally, although CUAVD is usually not associated with *CFTR* gene mutations [2], genetic counseling is prudent.

**Fig. 2:**
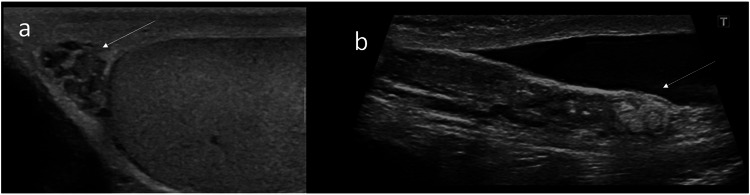
Epididymal findings in congenital absence of vas deferens in two patients. **a** Only a small, inhomogeneous epididymal head was found. Neither the epididymal body and tail nor the vas deferens were found. **b** Epididymal body with sudden interruption ending abruptly including a dilated tube with echoic content (long arrow). Neither the epididymal tail nor the vas deferens were found

In the case of CAVD, the epididymis may be present and dilated, often with tubular ectasia, or rarely may be absent [2]. In both cases, the epididymal head is always detectable [2], and can be either dilated or small (Fig. 2).

Pelvic MRI can be used when the US study of the vas deferens is doubtful/inconclusive or to evaluate the intra-abdominal course of the vas deferens (poorly explorable with US), as well as to investigate the organs of the prostate-vesicular region when suprapubic or transrectal US are doubtful/inconclusive assessing abnormalities related to suspected OA and/or low seminal volume and pH [113–115].

*Recommendation 12: In infertile men, vas deferens US investigation is recommended, especially when physical examination is unreliable. Detection of CBAVD leads to proven OA diagnosis. Detection of CUAVD does not exclude fertility. Detection of CBAVD or CUAVD should lead to an extension of the US examination for evaluation of the SV and kidneys*.

*Recommendation 13: In infertile men, pelvic MRI can be used when the US study of the vas deferens is doubtful/inconclusive or to evaluate the intra-abdominal course of the vas deferens, as well as to investigate the prostate-vesicular region when suprapubic or transrectal US are doubtful/inconclusive assessing abnormalities related to suspected OA and/or low seminal volume and pH*.

##### Epididymis

**Fig. 3:**
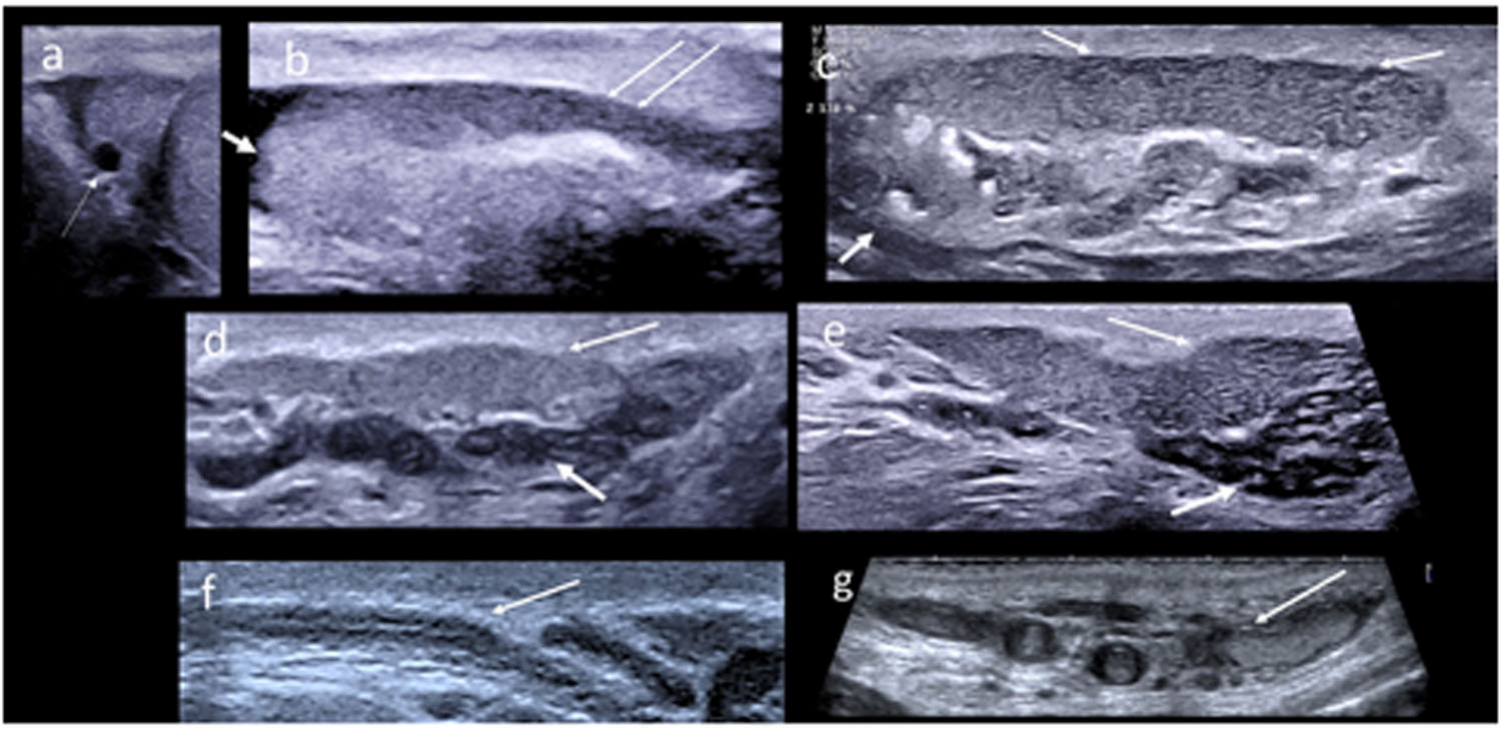
Normal and obstructive signs of the epididymis and vas deferens. **a** Normal epididymal head with small cyst (arrow); **b** normal epididymal head (small arrow) and body (thin arrow); **c** tubular ectasia of the epididymal body (thin arrows), echoic foci of the epididymal head (small arrow); **d** normal epididymal body and tail (thin arrow), and winding first part of the vas deferens (small arrow); **e** tubular ectasia of the epididymis (long arrow) and of the vas deferens (small arrow); **f** normal vas deferens in its distal scrotal part, with a linear path and a thin lumen (arrow); **g** tubular ectasia of the vas deferens with echoic stagnant sperm (arrow)

The scrotal US plays a key role in investigating abnormalities of epididymal size, echopattern, and vascularization, which, alone or combined, can suggest different diagnoses [2, 7, 116–120]. In subjects with scrotal pain or prostatitis-like symptoms, epididymal dilation with hypervascularization suggests inflammation [2, 7, 116–120]. A dilated epididymis associated with echopattern abnormalities may also represent the outcome of a past infection/inflammation in currently asymptomatic patients [2, 7, 117–122]. In subjects with obstructive azoo-/oligo-spermia, epididymal enlargement with tubular ectasia may suggest, as an indirect sign, post-testicular obstruction, at the epididymal [123] (Fig. 3), vas deferens [124] or prostatic level [125, 126], and the latter may be further investigated by extending US to the prostate-vesicular region. Current or previous epididymal inflammation or partial obstruction has been associated with sperm abnormalities [127, 128]. Of note, only a proven bilateral epididymis obstruction can diagnose proximal OA. However, US can only suggest, but not prove, the presence of a complete epididymal obstruction. The scrotal US also allows the assessment of epididymal nodules [118–121], frequently represented by cysts, which have no proven role in OA [110]. Rarely, underlying benign or malignant tumors may be identified [117–120].

*Recommendation 14: In infertile men, epididymis investigation with scrotal US is indicated, to detect indirect signs suggesting obstruction and/or inflammation, possibly exerting a negative impact on sperm parameters. Cysts or nodules should be investigated: they have no proven role in infertility but may be relevant for general male health*.

#### Scrotal emergencies and male infertility: role of imaging

Emergencies are medical conditions requiring prompt treatment to minimize the likelihood of loss of organ structure or function, and in rare cases, of the patient’s life. Scrotal emergencies include different entities, such as testicular torsion, trauma, epididymo-orchitis, and someway, malignancies [129]. The role of these acute conditions and their chronic outcomes in male infertility has been assessed by relatively few studies, reporting various transient or long-lasting negative effects on sperm and, rarely, hormonal parameters, according to the different conditions, severity of the condition, extension of the testicular damage, rapidity and effectiveness of medical or surgical intervention [2, 116, 127–131]. Besides medical history, sometimes pathognomonic, and clinical signs and symptoms, grayscale and color-Doppler US play a key role in several conditions, eventually supported by contrast-enhanced US and sonoelastography as problem-solving modalities in some equivocal cases [2, 7, 65, 129, 132–135]. Scrotal MRI is rarely needed for the assessment of acute scrotum or scrotal trauma in cases of non-diagnostic US findings [136]. Scrotal emergencies are usually characterized by scrotal acute pain and swelling [2, 65, 129, 132, 133]. At color-Doppler US, testicular torsion is usually characterized by the absence of blood flow in the symptomatic testis, while epididymo-orchitis by the presence of enhanced blood flow in the affected testis and epididymis [2, 7, 65, 129]. Approximately 10% of patients with a testicular malignancy present with pain, although the typical presentation of a testicular cancer is painless, and a small or large nodule, solid or mixed, with internal vascularization can be detected by color-Doppler US [2, 7, 65, 129]. Testicular trauma can be blunt, penetrating, or degloving, and show typical US features in case of a hematoma, contusion, fracture, or rupture, with or without hematocele, which may change over time [132, 133]. The aforementioned conditions may also lead to testicular infarction, detectable as a hypoechoic wedge-shaped lesion with absent internal vascularization and a peripheral rim of low vascular signal [2, 7, 65, 129, 132, 133].

*Recommendation 15: In scrotal emergencies, the radiologist should evaluate the medical history and clinical signs and symptoms of the patient, and perform US to contribute to the diagnosis of testicular torsion, trauma, epididymo-orchitis or malignancy, which could exert a transient or long-lasting negative effect on sperm parameters and male fertility. Scrotal MRI is rarely needed in cases of non-diagnostic US findings*.

*Recommendation 16: In infertile men, the radiologist should investigate the history of scrotal emergencies/acute scrotum to detect and/or understand related testicular US abnormalities*.

## Conclusions

The ESUR-SPIWG recommendations on scrotal imaging in the evaluation of male infertility are herein reported and discussed.

## Supplementary information

supplementary material

## Abbreviations

EAA: European Academy of Andrology
EAU: European Association of Urology
ESUR: European Society of Urogenital Radiology
GoR: Grade
GRADE: Grading of Recommendations Assessment, Development, and Evaluation
LoE: Level of evidence
MGT: Male genital tract
MRI: Magnetic resonance imaging
NOA: Non-obstructive oligo-/azoo-spermia
OA: Obstructive oligo-/azoo-spermia
SPIWG: Scrotal and Penile Imaging Working Group
TI: Testicular inhomogeneity
TML: Testicular microlithiasis
TV: Testicular volume
US: Ultrasound
