# Supplementary material for: The role of the radiologist in the evaluation of male infertility: recommendations of the European Society of Urogenital Radiology-Scrotal and Penile Imaging Working Group (ESUR-SPIWG) for scrotal imaging
Source: Eur Radiol. 2024 Jul 31;35(2):752–66. doi: 10.1007/s00330-024-10964-5 (PMC11782349; doi:10.1007/s00330-024-10964-5)
Supplement: Supplementary file 1 — supplementary material [file 330_2024_10964_MOESM1_ESM.pdf]

Supplementary Table S1 - Oxford Centre for Evidence-Based Medicine 2011 Levels of Evidence (Diagnosis)

Level	
1	Systematic review of cross sectional studies with consistently applied reference standard and blinding
2	Individual cross sectional studies with consistently applied reference standard and blinding
3	Non-consecutive studies, or studies without consistently applied reference standards
4	Case-control studies, or "poor or non-independent reference standard*
5	Mechanism-based reasoning

Source: OCEBM Levels of Evidence Working Group. "The Oxford 2011 Levels of Evidence". Oxford Centre for Evidence-Based Medicine. http://www.cebm.net/index.aspx?o=5653. Accessed: 25 February 2024

Supplementary Table S2 – Grading of Recommendations Assessment, Development and Evaluation (GRADE)

Code	Quality of Evidence	Definition
A	High	Several high-quality studies with consistent results<br>In special cases: one large, high-quality multi-centre trial
B	Moderate	One high-quality study<br>Several studies with some limitations
C	Low	One or more studies with severe limitations
D	Very Low	Expert opinion<br>No direct research evidence<br>One or more studies with very severe limitations

Source: https://www.essentialevidenceplus.com/product/ebm_loe.cfm?show=grade. Accessed 25 February 2025
